# *CYP1A1*和*GSTM1*基因多态性及其对个体肺癌易感性的联合效应：*meta*分析

**DOI:** 10.3779/j.issn.1009-3419.2011.08.05

**Published:** 2011-08-20

**Authors:** 程 李, 智华 尹, 宝森 周

**Affiliations:** 110001 沈阳，中国医科大学公共卫生学院流行病学教研室，辽宁省高校肿瘤病因与预防重点实验室，循证医学中心 Department of Epidemiology, School of Public Health, China Medical University; The Key Laboratory of Cancer Control in Liaoning Province; China Medical University Center for Evidence Based Medicine, Shenyang 110001, China

**Keywords:** CYP1A1, GSTM1, 基因多态性, 肺肿瘤, *meta*分析, Cytochrome P450A1 (CYP1A1), Glutathione S-transferase M1 (GSTM1), Genetic polymorphism, Lung neoplasms, *meta*-analysis

## Abstract

**背景与目的:**

谷胱甘肽转移酶M1(glutathione S-transferase M1, GSTM1)和细胞色素P4501A1(cytochrome P450A1, CYP1A1)均存在基因多态性，并且对肺癌发病风险有一定的影响，两者联合作用对肺癌发病风险的影响尚无确切定论。本研究旨在探讨*CYP1A1*和*GSTM1*基因多态性及其联合效应与肺癌危险性的关系。

**方法:**

在PubMed数据库、EMBASE数据库、中国生物医学文献数据库(china biology medicine, CBM)和中国知识基础设施工程数据库(china national knowledge infrastructure, CNKI)中查询文献，时间范围从各数据库建库至2011年3月。使用STATA 10软件进行*meta*分析统计，对于每篇入选的文献均计算肺癌发生危险性调整混杂因素后优势比(odd ratio, OR)及其95%置信区间(confidence interval, CI)。

**结果:**

15篇文献最终被纳入本次研究。*Meta*分析显示*GSTM1*基因缺失时*CYP1A1*基因IIe/Val位点为纯合突变型时肺癌发病风险明显高于杂合型与纯合突变型联合，总体OR分别为3.18(95%CI: 1.27-7.98)和1.45(95%CI: 1.08-1.94)。*GSTM1*基因缺失时*CYP1A1*基因MspI位点为纯合突变型时肺癌发病风险也高于杂合型与纯合突变型联合，总体OR分别为1.90(95%CI: 1.00-3.58)和1.57(95%CI: 1.23-2.00)。

**结论:**

CYP1A1和GSTM1基因多态性联合作用增加了单个基因多态性发生肺癌的危险性。CYP1A1纯合突变型基因对人群肺癌易感性的影响明显大于野生型和杂合突变型。

## Abstract (en)

**Background and objective:**

Cytochrome P450A1 (*CYP1A1*) gene and glutathione S-transferase M1 (*GSTM1*) gene both have single nucleotide polymorphisms and effects on lung cancer. Currently, however, the risk of lung cancer due to the *CYP1A1* and *GSTM1* genes has no clear evidence. In this present study, we propose to research the combined effects of *CYP1A1* gene and *GSTM1* gene polymorphism and their risks to lung cancer.

**Methods:**

We conducted the study at different research areas and using various database, including PubMed, Embase, China Biology Medicine (CBM) and China National Knowledge Infrastructure (CNKI) last March 31, 2011. We calculated the adjusted odds ratio (OR) and 95% confidence interval (CI) for lung cancer in each study. Using STATA 10, a statistical program, we summarized the calculated estimates for the adjusted ORs and performed a *meta*-analysis.

**Results:**

The *meta*-analysis includes 15 research studies. The CYP1A1 IIe/Val genotype which carries a homozygous mutant type has a higher chance of risk to lung cancer than that which carries a homozygous mutant type and a heterozygous type when the GSTM1 carries a null genotype. As a result, OR was 3.18 (95%CI: 1.27-7.98), 1.45 (95%CI: 1.08-1.94), respectively. Meanwhile, the same conclusion was obtained for the CYP1A1 MspI genotype. The overall OR was 1.90 (95%CI: 1.00-3.58), 1.57 (95%CI: 1.23-2.00), respectively.

**Conclusions:**

We discovered through our *meta*-analysis that the combined effects of *CYP1A1* gene and *GSTM1* gene polymorphism are significantly associated with an increased risk to lung cancer. We also found that homozygous mutant genotype of CYP1A1 has a higher chance of risk to lung cancer than the homozygous or heterozygous genotype.

根据世界卫生组织(world heath organization, WHO) 2003年公布的资料显示，肺癌无论是发病率还是死亡率均居全球癌症的首位^[1]^。谷胱甘肽转移酶(glutathione S-transferase, GST)是一类重要的外源性化学物代谢酶，主要催化外援代谢物的亲电子中心与还原型谷胱甘肽的结合反应，从而降低外源化合物的毒性及致癌性^[2]^。谷胱甘肽转移酶M1(glutathione S-transferase M1, GSTM1)存在等位基因缺失的多态性，GSTM1基因多态性可以改变相应的酶激活或灭活异源底物的能力，可能对个体暴露后所致肺癌的易感性产生影响^[3-6]^。细胞色素P450酶(cytochrome P450A1, CYP1A1)是Ⅰ相代谢酶，是代谢内外源性化合物的重要酶系，其编码的芳烃羟化酶参与致癌物进入体内的第一阶段氧化激活反应，将外源性无活性的前致癌物激活转变为有活性的亲电子化合物，与细胞内大分子DNA或蛋白质结合形成加合物，使DNA发生突变，导致某些癌基因的激活或抑制，最终导致癌变^[7-9]^。近年来，有多篇关于这两个基因联合作用的文章，并且重点研究了CYP1A1两个位点Ⅱe/Val和MspI w1/m1的多态性。但是并没有CYP1A1的Ⅱe/Val位点与GSTM1联合基因型分析的*meta*分析，在之前的研究中也没有涉及过CYP1A1的MspI位点与GSTM1联合基因型分析的*meta*分析。本研究旨在通过*meta*分析探讨CYP1A1两个位点分别和*GSTM1*基因多态性的联合效应与肺癌发病风险的关系。

## 1 材料与方法

### 1.1 文献入选标准

① 肺癌；②CYP1A1 Ⅱe/Val位点和*GSTM1*基因多态性与肺癌易感性病例对照研究和队列研究；③CYP1A1 MspI位点和GSTM1基因多态性与肺癌易感性病例对照研究和队列研究；④所有研究中以下因素要有明确数据：作者、文献发表年份、肺癌病例的基本特征(年龄分布、性别比例、吸烟与否)、对照人群的基本特征(年龄分布、性别比例、吸烟与否)、病例与对照各类基因型分别的人数、OR值与调整后OR值；⑤当多个研究使用同一数据时纳入最近的文献或是包含信息更多的文献；⑥各基因多态性的鉴定均采用等位基因特异性和多重差别聚合酶链反应(polymerase chain reaction, PCR)方法。

### 1.2 文献排除标准

① 同时伴随其他恶性肿瘤；②对照设立不正确；③无原始数据。

### 1.3 检索策略

以“CYP1A1”、“cytochrome P450A1”、“GSTM1”、“ glutathione S-transferase M1”、“lung cancer”、“NSCLC”、“lung carcinoma”和“polymorphism”为关键词，在PubMed数据库和EMBASE数据库中查询文献，以“CYP1A1”、“细胞色素P4501A1”、“GSTM1”、“谷胱甘肽转移酶M1”、“肺癌”、“非小细胞肺癌”、“多态性”为关键词中国生物医学文献数据库(china biology medicine, CBM)和中国知识基础设施工程数据库(china national knowledge infrastructure, CNKI)中查询文献，时间范围从各数据库建库至2011年3月。纳入文献包括英文文献和中文文献。

### 1.4 文献筛选和资料提取

根据事先制定的纳入标准两位研究者交叉核对纳入研究结果，缺乏的数据通过邮件与作者联系予以补充。提取数据的主要内容包括作者、文献发表时间、研究对象种族、研究对象一般特征、研究方法等。

### 1.5 质量评价

采用Newcastle-Ottawa Scale(NOS)标准进行质量评价^[10]^：①病例组和对照组研究对象选择(4分)：病例定义、病例代表性、对照选择、对照定义；②病例组和对照组研究对象的可比性(2分)：首要因素、其他因素；③结果评价(3分)：暴露确证、同一确证、不回应率。若每一条为否时，赋值0，若为是，赋值1。

### 1.6 统计分析方法

使用STATA 10软件进行统计分析。对于每个研究都要计算调整混杂因素后的OR值与95%CI并计算总体的OR值与95%CI。对纳入研究结果间的异质性分析采用χ^2^检验。当*P* > 0.05和*I*^2^ < 50%时使用固定效应模型进行分析，若存在统计学异质性，即*P* < 0.05，*I*^2^ > 50%时分析异质性来源，确定是否能采用随机效应模型。当研究存在明显异质性时，只对其进行描述性分析。通过漏斗图与*Egger’s*检验评估发表偏倚。采用敏感性分析对结果稳定性进行检验。

## 2 结果

### 2.1 纳入研究概述

按照检索策略(图 1)，共检索到相关文献376篇，通过阅读标题、摘要进行初筛后纳入文献112篇。进一步阅读文献排除重复或不符合纳入标准的文献后剩下18篇文献。进行全文阅读后9篇关于CYP1A1 Ⅱe/Val位点与GSTM1联合基因型和肺癌易感性的病例对照研究的文献入选^[11-19]^，其中1篇因缺少原始数据而被排除^[19]^。9篇关于CYP1A1 w1/m1位点与*GSTM1*联合基因型和肺癌易感性的病例对照研究的文献入选^[20-28]^，其中1篇因原始数据不充分而被排除^[20]^，Li^[22]^与Li^[23]^使用相同原始数据，故排除前1篇。在剩下的15篇文献中，基因型的分布在所有研究的对照组中均符合*Hardy-Weinberg*遗传平衡定律。

**1:**
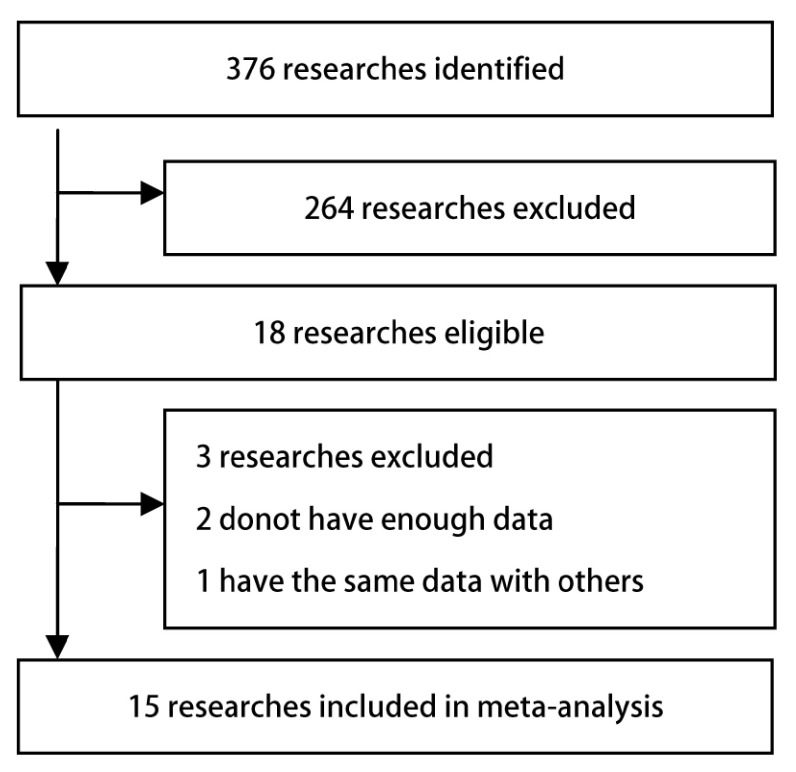
纳入研究流程图 Selection of trials

### 2.2 纳入研究的一般情况和质量评价

见表 1。15项研究经得分统计后除了Qu^[11]^和Hong^[24]^两项研究为B类研究，剩下13篇纳入文献均为A类研究，文献质量较好。表 2和表 3列出了CYP1A1 Ⅱe/Val位点和GSTM1联合基因型总体OR与肺癌易感性间的关系及CYP1A1 MspI w1/m1位点和GSTM1联合基因型总体OR与肺癌易感性间的关系。所有数据异质性检验*P* > 0.05，所以分析各个总体OR值均采用固定效应模型。

**1:** 纳入研究的一般情况和质量评价 The characteristics of included studies and quality assessment

Author	Year	No. of cases	No. of controls	NOS score				Level
				Selection	Comparability	Exposure	Summary	
Nakachi^[12]^	1993	85	170	3	2	1	6	A
Qu^[11]^	1998	180	179	3	1	1	5	B
Persson^[13]^	1999	75	119	3	2	1	6	A
Hayashi ^[15]^	2000	156	148	3	2	1	6	A
Xue^[14]^	2001	106	106	4	2	1	7	A
Zhang^[17]^	2002	65	60	3	2	1	6	A
Quinones^[16]^	2004	197	144	3	2	1	6	A
Nimura^[18]^	1997	91	137	3	2	1	6	A
Gu^[21]^	2004	180	224	3	2	2	7	A
Li^[23]^	2006	98	136	4	2	1	7	A
Hong^[24]^	1998	72	63	3	0	1	4	B
Jin^[25]^	2010	150	150	3	2	1	6	A
Zhu^[27]^	2010	160	160	4	2	2	8	A
Xia^[28]^	2008	58	116	3	2	2	7	A
Wang^[26]^	2006	91	86	3	2	2	7	A

**2:** CYP1A1 Ⅱe/Val位点和GSTM1联合基因型总体OR与肺癌易感性间的关系 Summary odds ratios relation of CYP1A1 Ⅱe/Val and GSTM1 combined polymorphism to lung cancer risk

Genotype	Case/Control	Heterogeneity test		OR (95%CI)	Hypothesis test		df	*Egger's* test	
		*Q*	*P*		*Z*	*P*		*t*	*P*
Ⅱe/Ⅱe ^*^GSTM1	539/716	11.02	0.201	1.28 (1.02-1.61)	2.45	0.014	8	2.19	0.080
Val/Val ^*^GSTM1	62/33	0.74	0.994	3.18 (1.27-7.98)	2.60	0.009	6	1.31	0.281
Ⅱe/Val & Val/Val ^*^GSTM1	416/347	9.75	0.283	1.45 (1.08-1.94)	3.07	0.002	8	1.05	0.342

**3:** CYP1A1 MspI w1/m1位点和GSTM1联合基因型总体OR与肺癌易感性间的关系 Summary odds ratios relation of CYP1A1 MspI w1/m1and GSTM1 combined polymorphism to lung cancer risk

Genotype	Case/Control	Heterogeneity test		OR (95%CI)	Hypothesis test		df	*Egger's* test	
		*Q*	*P*		*Z*	*P*		*t*	*P*
w1/w1^*^GSTM1	294/406	1.80	0.937	1.39 (1.02-1.90)	2.11	0.035	6	-1.00	0.423
m1/m1^*^GSTM1	97/84	0.88	0.927	1.90 (1.00-3.58)	1.97	0.049	4	-1.00	0.393
w1/m1 & ;m1/m1^*^GSTM1	528/534	4.14	0.657	1.57 (1.23-2.00)	3.59	0.000	6	-0.38	0.741

### 2.3 *meta*分析结果

CYP1A1 Ⅱe/Val位点与*GSTM1*联合基因型病例对照研究与肺癌危险性关系见表 4。CYP1A1为野生型Ⅱe/Ⅱe、纯合突变型Val/Val和杂合型与纯合突变型联合时，*GSTM1*基因缺失均增加肺癌发病风险，总体OR分别为1.28(95%CI: 1.02-1.61)、3.18(95%CI: 1.27-7.98)和1.45(95%CI: 1.08-1.94)。当CYP1A1为纯合突变型时增加肺癌发病风险明显高于其他类型突变。CYP1A1 MspI位点与*GSTM1*联合基因型病例对照研究与肺癌危险性关系见表 5。CYP1A1为野生型、纯合突变型和杂合型与纯合突变型联合时，*GSTM1*基因缺失增加肺癌发病风险均有统计学意义，总体OR分别为1.39(95%CI: 1.02-1.90)、1.90(95%CI: 1.00-3.58)、1.57(95%CI: 1.23-2.00)。

**4:** CYP1A1 Ⅱe/Val位点与GSTM1联合基因型病例对照研究与肺癌危险性关系 Case–control studies for CYP1A1 Ⅱe/Val and GSTM1 combined gene polymorphism and lung cancer risk

Author	Year	GSTM1 genotype	CYP1A1 Ⅱe/Ⅱe genotype			GSTM1 genotype	CYP1A1 Val/Val genotype			GSTM1 genotype	CYP1A1 Ⅱe/Val & Val/Val genotype		
			Case	Control	OR (95%CI)		Case	Control	OR (95%CI)		Case	Control	OR (95%CI)
Nakachi^[12]^	1993	+	19	57	1.00	+	1	3	1.00	+	14	29	1.00
		-	31	53	1.75 (0.89-3.47)	-	8	3	8.00 (0.58-110.27)	-	21	31	1.40 (0.60-3.27)
Nimura^[18]^	1997	+	27	45	1.00	+	7	5	1.00	+	15	29	1.00
		-	23	47	0.82 (0.41-1.63)	-	8	2	2.86 (0.42-19.65)	-	26	16	3.14 (1.30-7.58)
Qu^[11]^	1998	+	39	50	1.00	+	4	4	1.00	+	39	35	1.00
		-	59	56	1.35 (0.77-2.36)	-	6	3	2.00 (0.28-14.20)	-	43	38	1.02 (0.54-1.91)
Persson^[13]^	1999	+	17	20	1.00	+	3	4	1.00	+	10	20	1.00
		-	32	57	0.66 (0.30-1.44)	-	3	1	4.00 (0.27-60.32)	-	16	22	1.45 (0.54-3.94)
Hayashi^[15]^	2000	+	49	55	1.00	+	-	-	-	+	14	8	1.00
		-	71	76	1.05 (0.63-1.73)	-	-	-	-	-	22	9	1.40 (0.44-4.48)
Xue^[14]^	2001	+	27	42	1.00	+	4	2	1.00	+	21	25	1.00
		-	31	28	1.72 (0.85-3.48)	-	6	1	3.00 (0.20-45.24)	-	27	11	2.92 (1.18-7.26)
Zhang^[17]^	2002	+	8	21	1.00	+	4	3	1.00	+	16	12	1.00
		-	16	11	3.82 (1.25-11.69)	-	8	2	3.00 (0.35-25.87)	-	25	16	1.17 (0.44-3.11)
Quinones^[16]^	2004	+	29	41	1.00	+	-	-	-	+	53	23	1.00
		-	61	57	1.51 (0.83-2.75)	-	-	-	-	-	54	23	1.02 (1.08-2.03)
					Summary				Summary				Summary
		+			1.00	+			1.00	+			1.00
		-			1.28 (1.02-1.61)	-			3.18 (1.27-7.98)	-			1.45 (1.08-1.94)

**5:** CYP1A1 MspI位点与GSTM1联合基因型病例对照研究与肺癌危险性关系 Case–control studies for CYP1A1 MspI and GSTM1 combined gene polymorphism and lung cancer risk

Author	Year	GSTM1 genotype	CYP1A1 w1/m1 genotype			GSTM1 genotype	CYP1A1 m1/m1genotype			GSTM1 genotype	CYP1A1 w1/m1 & m1/m1genotype		
			Case	Control	OR (95%CI)		Case	Control	OR (95%CI)		Case	Control	OR (95%CI)
Hong^[24]^	1998	+	14	11	1.00	+	3	1	1.00	+	24	19	1.00
		-	21	15	1.10 (0.39-3.08)	-	4	2	1.71 (0.29-10.30)	-	26	18	1.14 (0.49-2.68)
Gu^[21]^	2004	+	25	47	1.00	+	-	-	-	+	54	75	1.00
		-	26	39	1.25 (0.63-2.51)	-	-	-	-	-	75	63	1.65 (1.02-2.68)
Wang^[26]^	2006	+	16	19	1.00	+	9	12	1.00	+	19	21	1.00
		-	16	16	1.19 (0.45-3.10)	-	27	14	0.67 (0.04-11.29)	-	40	35	2.25 (1.14-4.46)
Li^[23]^	2006	+	11	39	1.00	+	3	4	1.00	+	27	37	1.00
		-	14	32	1.55 (0.62-3.88)	-	9	7	1.71 (0.62-4.71)	-	46	28	1.26 (0.59-2.72)
Xia^[28]^	2008	+	7	21	1.00	+	1	6	1.00	+	17	34	1.00
		-	10	19	1.58 (0.50-4.98)	-	4	12	2.00 (0.18-22.06)	-	24	42	1.14 (0.53-2.46)
Zhu^[27]^	2010	+	29	38	1.00	+	15	14	1.00	+	38	50	1.00
		-	26	30	1.14 (0.56-2.32)	-	22	12	2.57 (0.87-7.56)	-	67	42	1.22 (0.62-2.39)
Jin^[25]^	2010	+	27	40	1.00	+	-	-	-	+	28	31	1.00
		-	52	40	1.93 (1.02-3.65)	-	-	-	-	-	43	39	2.10 (1.19-3.72)
					Summary				Summary				Summary
					1.00				1.00				1.00
					1.39 (1.02-1.90)				1.90 (1.00-3.58)				1.57 (1.23-2.00)

### 2.4 发表偏倚

对纳入的所有文献进行了漏斗图分析(图 2)，结果显示图形对称性良好。*Egger's*检验结果(表 2，表 3，图 3，图 4)显示无明显发表偏倚。

**2:**
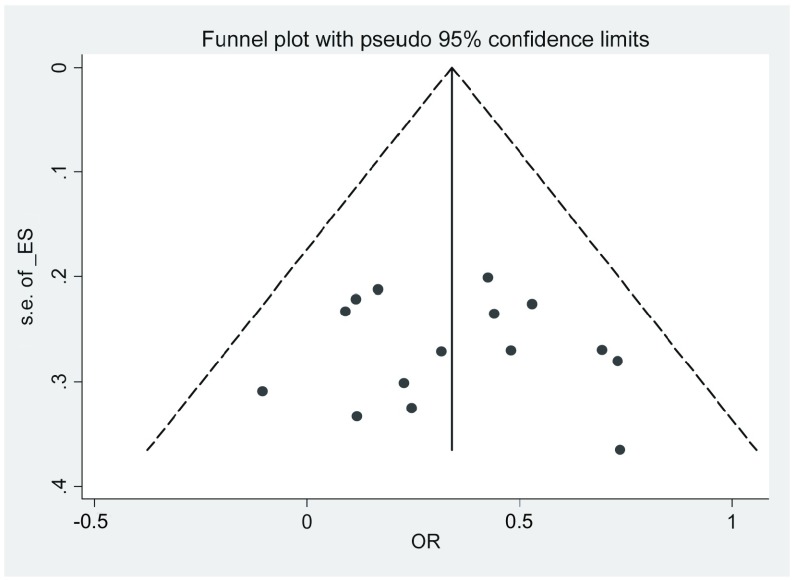
漏斗图 Funnel plot

**3:**
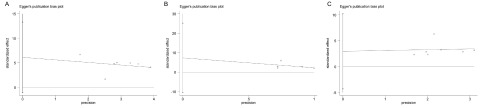
CYP1A1 Ⅱe/Val位点与GSTM1联合基因型病例对照研究与肺癌危险性关系*Egger's*检验。A：为CYP1A1 Ⅱe/Ⅱe和GSTM1联合基因型；B：CYP1A1 Val/ Val和GSTM1联合基因型；C：CYP1A1 Ⅱe/Val & Val/Val和GSTM1联合基因型。 Egger's test of the summary odds ratio coefficients on the association between CYP1A1 Ⅱe/Val and GSTM1 combined gene polymorphism and lung cancer risk. A: CYP1A1 Ⅱe/Ⅱ and GSTM1 combined; B: CYP1A1 Val/Val and GSTM1 combined; C: CYP1A1 Ⅱe/Val & Val/Val and GSTM1combined.

**4:**
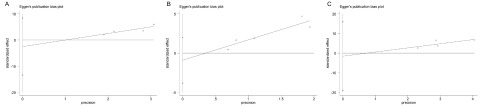
CYP1A1 MspI位点与GSTM1联合基因型病例对照研究与肺癌危险性关系*Egger's*检验。A：分别为CYP1A1 MspI w1/w1和GSTM1联合基因型；B：CYP1A1 MspI m1/m1和GSTM1联合基因型；C：CYP1A1 MspI w1/m1 & m1/m1和GSTM1联合基因型。 Egger's test of the summary odds ratio coefficients on the association between CYP1A1 MspI and GSTM1 combined gene polymorphism and lung cancer risk. A: CYP1A1 MspI w1/w1 and GSTM1 combined; B: CYP1A1 MspI m1/m1 and GSTM1 combined; C: CYP1A1 MspI w1/m1 & m1/m1 and GSTM1 combined.

### 2.5 敏感性分析

见图 5。结果显示依次排除各篇文章进行分析，结果显示C YP1A1 Ⅱe/Ⅱe和GSTM1联合基因型、CYP1A1 Val/Val和GSTM1联合基因型、CYP1A1 Ⅱe/Val & Val/Val和GSTM1联合基因型的OR值分别为1.45 (95%CI: 1.21-1.70)、3.67(95%CI: 2.62-4.73)和1.39 (95%CI: 1.07-1.71)，与*meta*分析所得OR非常接近，说明研究稳定性良好。图 6分别显示了CYP1A1 MspI w1/w1和GSTM1联合基因型、CYP1A1 MspI m1/m1和GSTM1联合基因型、CYP1A1 MspI w1/m1 & m1/m1和GSTM1联合基因型OR值分别为1.53(95%CI: 1.14-1.92)、1.98(95%CI: 1.34-2.62)和1.61(95%CI: 1.30-1.93)，研究稳定性良好。

**5:**
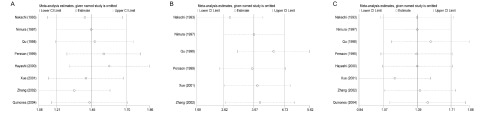
CYP1A1 Ⅱe/Val位点与GSTM1联合基因型病例对照研究与肺癌危险性关系敏感性分析。A：CYP1A1 Ⅱe/Ⅱe和GSTM1联合基因型；B：CYP1A1 Val/Val和GSTM1联合基因型；C：CYP1A1 Ⅱe/Val & Val/ Val和GSTM1联合基因型。 Influence analysis of the summary odds ratio coefficients on the association between CYP1A1 Ⅱe/Val and GSTM1 combined gene polymorphism and lung cancer risk. A: CYP1A1 Ⅱe/Ⅱe and GSTM1 combined; B: CYP1A1 Val/Val and GSTM1 combined; C: CYP1A1 Ⅱe/Val & Val/Val and GSTM1combined.

**6:**
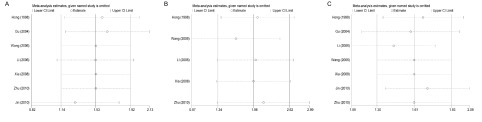
CYP1A1 MspI位点与GSTM1联合基因型病例对照研究与肺癌危险性关系敏感性分析。A：CYP1A1 MspI w1/w1和GSTM1联合基因型；B：CYP1A1 MspI m1/m1和GSTM1联合基因型；C：CYP1A1 MspI w1/m1 & m1/m1和GSTM1联合基因型。 Influence analysis of the summary odds ratio coefficients on the association between CYP1A1 MspI and GSTM1 combined gene polymorphism and lung cancer risk. A: CYP1A1 MspI w1/w1 and GSTM1 combined; B: CYP1A1 MspI m1/m1 and GSTM1 combined; C: CYP1A1 MspI w1/m1 & m1/m1 and GSTM1 combined.

## 3 讨论

之前的研究并未有关于C Y P 1 A 1的I Ie / Va l位点与GSTM1联合基因型分析的*meta*分析，以及CYP1A1的MspI位点与GSTM1联合基因型分析的*meta*分析，本研究旨在利用*meta*分析探讨CYP1A1两个位点分别和GSTM1基因多态性的联合效应与肺癌发病风险关系。

在中国人群的研究中Gu^[21]^、Song^[29]^发现至少携带一个变异等位基因的个体明显增加肺癌发病风险。而Persson^[13]^、Wang^[30]^认为CYP1A1变异等位基因与肺癌发病风险没有直接关系。在高加索人群中有些研究^[31, 32]^并未显示*CYP1A1*基因变异与肺癌发病风险之间的关系。许多研究^[14, 21]^表明*GSTM1*基因缺失多态性增加肺癌的发病风险。本次研究结果表明携带CYP1A1 Ⅱe/Val & Val/Val基因型、携带CYP1A1 MspI w1/m1 & m1/m1基因型、携带GSTM1缺失基因型均增加了肺癌发病风险。携带CYP1A1 Ⅱe/Val & Val/Val和GSTM1-的联合基因型的个体罹患肺癌的危险度高于携带单一突变易感基因型个体，总体OR分别为1.45(95%CI: 1.08-1.94)和1.28(95%CI: 1.02-1.61)。携带CYP1A1 MspI w1/m1 & m1/m1基因型和GSTM1-联合基因型的个体同样肺癌发病风险高于携带单一基因突变型的个体，总体OR分别为1.57(95%CI: 1.23-2.00)和1.39 (95%CI: 1.02-1.90)。本次研究显示CYP1A1相互作用增加了个体肺癌发病的危险性。

GSTM1基因多态性可以改变相应的酶激活或灭活异源底物的能力，可能对个体暴露后所致肺癌的易感性产生影响。*CYP1A1*基因将外源性无活性的前致癌物激活转变为有活性的亲电子化合物，与细胞内大分子DNA或蛋白质结合，形成加合物，使DNA发生突变，导致某些癌基因的激活或抑制，最终导致癌变。有研究^[14]^显示人群中携带CYP1A1 Ⅱe/Val与GSTM1-联合基因型个体的频率约是CYP1A1 Val/Val与GSTM1联合基因型个体的4.5倍。在北欧人群的研究^[16]^中也提示CYP1A1 Ⅱe/Val或MspI遗传多态性中杂合子对人群的肺癌危险有较大的作用。但是在我们的研究中显示出CYP1A1 Ⅱe/Val遗传多态性中纯和突变型基因对人群的肺癌易感性的影响明显大于野生型和杂合突变型。这可能是由于本研究纳入的多是亚洲人群，由于不同种族基因突变频率不同导致。近年来关注CYP1A1的MspI位点研究增多，许多研究^[20-23]^表明此位点多态性影响肺癌发生易感性，但也有研究^[24]^显示此位点与肺癌易感性关系并无统计学意义。CYP1A1的MspI位点与GSTM1联合基因突变多态性明显增加了肺癌的易感性，*CYP1A1*基因这两个位点之间的相互作用应在今后进行更多的研究。

在CYP1A1、GSTM1分别与肺癌易感性关系的研究中，已对性别、吸烟与否和病理分型进行了亚组分析，但CYP1A1与GSTM1基因联合作用的研究并不多也未有原始资料对其进行各类的亚组分析，今后应进行更深入的研究。

## References

[b1] 1Lu ZY, Zhong NS ed. Internal Medicine. 7th ed. Beijing: People Medical Publishing House, 2009. 123-134.陆再英, 终南山主编. 内科学. 第7版. 北京: 人民卫生出版社, 2009. 123-134.

[2] Frova C (2006). Glutathione transferases in the genomics era: new insights and perspectives. Biomol Eng.

[3] Hayes JD, Pulford DJ (1995). The glutathione S-transferase supergene family: regulation of GST and the contribution of the isoenzymes to cancer chemoprotection and drug resistance. Crit Rev Biochem Mol Biol.

[4] Houlston RS (1999). Glutathione S-transferase M1 status and lung cancer risk: a *meta*-analysis. Cancer Epidemiol Biomarkers Prev.

[5] McWilliams JE, Sanderson BJ, Harris EL (1995). Glutathione S-transferase M1 (GSTM1) deficiency and lung cancer risk. Cancer Epidemiol Biomarkers Prev.

[6] Benhamou S, Lee WJ, Alexandrie AK (2002). *Meta*- and pooled analyses of the effects of glutathione S-transferase M1 polymorphisms and smoking on lung cancer risk. Carcinogenesis.

[7] Omiecinski CJ, Redlich CA, Costa P (1990). Induction and developmental expression of cytochrome P450IA1 messenger RNA in rat and human tissues: detection by the polymerase chain reaction. Cancer Res.

[8] Landi MT, Bertazzi PA, Shields PG (1994). Association between CYP1A1 genotype, mRNA expression and enzymatic activity in humans. Pharmacogenetics.

[9] Drakoulis N, Cascorbi I, Brockmoller J (1994). Polymorphisms in the human *CYP1A1* gene as susceptibility factors for lung cancer: exon-7 mutation (4889 A to G), and a T to C mutation in the 3'-flanking region. Clin Investig.

[b10] 10Wells GA, Shea B, O'Connell D, et al. The Newcastle-Ottawa Scale (NOS) for assessing the quality if nonrandomized studies in *meta*-analyses. Available from: URL: http: //www. ohri. ca/programs/clinical_epidemiology/oxford. htm [cited 2009 Oct 19].

[11] Qu YH, Shi YB, Zhong LJ (1998). The genotypes of cytochrome P4501A1 and GSTM1 in Non-smoking Female Lung Cancer. Tumor.

[12] Nakachi K, Imai K, Hayashi S (1993). Polymorphisms of CYP1A1 and glutathione S-T ransfease genes associated with susceptibility to lung cancer in elation to cigarette dose in a Japanese population. Cancer Res.

[13] Persson I, Johansson I, Lou YC (1999). Genetic polymorphism of xenobiotic *meta*bolizing enzymes among Chinese lung cancer patients. Int J Cancer.

[14] Xue KX, Xu L, Chen SQ (2001). Polymorphisms of the *CYP1A1* and *GSTM1* genes and their combined effects on individual susceptibility to lung cancer in a Chinese population. Chin J Med Genet.

[15] Hayashi S, Watanabe J, Kawajiri K (1992). High susceptibility to lung cancer analyzed in terms of combined genotypes of P450IA1 and Mu-class glutathione S-transferase genes. Jpn J Cancer Res.

[16] Quinones L, Lucas D, Godoy J (2001). *CYP1A1*, *CYP2E1* and *GSTM1* genetic polymorphisms. The effect of single and combined genotypes on lung cancer susceptibility in Chilean people. Cancer Lett.

[17] Zhang LZ, Wang X, Hao XZ (2002). Relationship between susceptibility to lung cancer and genetic polymorphism in P4501A1, GSTM1. Chin J Clin Oncol.

[18] Nimura Y, Yokoyama S, Fujimori M (1997). Genotyping of the *CYP1A1* and *GSTM1* genes in esophageal carcinoma patients with special reference to smoking. Cancer.

[19] Cote ML, Yoo W, Wenzlaff AS (2009). Tobacco and estrogen metabolic polymorphisms and risk of non-small cell lung cancer in women. Carcinogenesis.

[20] Chang FH, Hu TM, Wang G (2006). Relationship between *CYP1A1* and *GSTM1* genetic polymorphisms and lung cancer susceptibility in population of Inner Mongolia. Chin J Lung Cancer.

[21] Gu YF, Zhang SC, Lai BT (2004). Relationship between genetic polymorphism of *meta*bolizing enzymes and lung cancer susceptibility. Chin J Lung Cancer.

[b22] 22Li Y. Cytochrome P450 1A1 and Glutathione S-transferase M1 polymorphisms and susceptibility to lung cancer. Zhengzhou University, 2004.李英. 细胞色素P4501A1和谷胱甘肽硫转移酶M1基因多态性与肺癌易感性关系研究. 郑州大学, 2004.

[23] Li Y, Chen J, He X (2006). *CYP1A1* and *GSTM1* polymorphisms and susceptibility to lung cancer. J Zhengzhou University (Medical Science).

[24] Hong YS, Chang JH, Kwon OJ (1998). Polymorphism of the CYP1A1 and glutathione-S-transferase gene in Korean lung cancer patients. Exp Mol Med.

[25] Jin Y, Xu H, Zhang C (2010). Combined effects of cigarette smoking, gene polymorphisms and methylations of tumor suppressor genes on non small cell lung cancer: a hospital-based case-control study in China. BMC Cancer.

[26] Wang DQ, Chen SD, Wang BG (2006). A case control study on relationship between lung cancer and genetic polymorphism of *CYP1A1*, *CYP2E1* and *GSTM1* in Han Nationality in Guangzhou area. Bulletin of Chinese Cancer.

[27] Zhu XX, Hu CP, Gu QH (2010). CYP1 A1 polymorphisms, lack of glutathione S-transferase M1 (GSTM1), cooking oil fumes and lung cancer risk in nonsmoking women. Chin J Tuberc Respir Dis.

[28] Xia Y, Sun QF, Shang B (2006). Polymorphisms of the cytochrome P450 and glutathion s-transferase genes associated with lung cancer susceptibility for the residents in high radon-exposed area. Chin J Radiol Med Prot.

[29] Song N, Tan W, Xing D (2001). CYP 1A1 polymorphism and risk of lung cancer in relation to tobacco smoking: a case-control study in China. Carcinogenesis.

[30] Wang BG, Zhou WP, Li ZB (2004). A case control study on the impact of CYP450 MSPI and GST-M1 polymorphisms on the risk of lung cancer. Chin J Oncol.

[31] Alexandrie AK, Nyberg F, Warholm M (2004). Influence of CYP1A1, GSTM1, GSTT1, and NQO1 genotypes and cumulative smoking dose on lung cancer risk in a Swedish population. Cancer Epidemiol Biomarkers Prev.

[32] Hung RJ, Boffetta P, Brockmoller J (2003). CYP1A1 and GSTM1 genetic polymorphisms and lung cancer risk in Caucasian non-smokers: a pooled analysis. Carcinogenesis.

